# Downscaling land‐use data to provide global 30″ estimates of five land‐use classes

**DOI:** 10.1002/ece3.2104

**Published:** 2016-03-30

**Authors:** Andrew J. Hoskins, Alex Bush, James Gilmore, Tom Harwood, Lawrence N. Hudson, Chris Ware, Kristen J. Williams, Simon Ferrier

**Affiliations:** ^1^ CSIRO Land and WaterCanberraACT2601Australia; ^2^ Department of Life SciencesNatural History MuseumCromwell RoadLondonSW7 5BDUK

**Keywords:** Constrained optimization, global change, land cover, land use, landscape modification, statistical downscaling

## Abstract

Land‐use change is one of the biggest threats to biodiversity globally. The effects of land use on biodiversity manifest primarily at local scales which are not captured by the coarse spatial grain of current global land‐use mapping. Assessments of land‐use impacts on biodiversity across large spatial extents require data at a similar spatial grain to the ecological processes they are assessing. Here, we develop a method for statistically downscaling mapped land‐use data that combines generalized additive modeling and constrained optimization. This method was applied to the 0.5° Land‐use Harmonization data for the year 2005 to produce global 30″ (approx. 1 km^2^) estimates of five land‐use classes: primary habitat, secondary habitat, cropland, pasture, and urban. The original dataset was partitioned into 61 bio‐realms (unique combinations of biome and biogeographical realm) and downscaled using relationships with fine‐grained climate, land cover, landform, and anthropogenic influence layers. The downscaled land‐use data were validated using the PREDICTS database and the geoWiki global cropland dataset. Application of the new method to all 61 bio‐realms produced global fine‐grained layers from the 2005 time step of the Land‐use Harmonization dataset. Coarse‐scaled proportions of land use estimated from these data compared well with those estimated in the original datasets (mean *R* ^2^: 0.68 ± 0.19). Validation with the PREDICTS database showed the new downscaled land‐use layers improved discrimination of all five classes at PREDICTS sites (*P *<* *0.0001 in all cases). Additional validation of the downscaled cropping layer with the geoWiki layer showed an *R* ^2^ improvement of 0.12 compared with the Land‐use Harmonization data. The downscaling method presented here produced the first global land‐use dataset at a spatial grain relevant to ecological processes that drive changes in biodiversity over space and time. Integrating these data with biodiversity measures will enable the reporting of land‐use impacts on biodiversity at a finer resolution than previously possible. Furthermore, the general method presented here could be useful to others wishing to downscale similarly constrained coarse‐resolution data for other environmental variables.

## Introduction

Across the globe, anthropogenic use of the environment has led to changes in the quality and health of ecosystems (Foley et al. 2005). The clearance, modification, and fragmentation of natural habitat for human use has been a major driver of biodiversity loss at local, regional, and global scales (Chapin et al. 2000; Sala et al. 2000; Fischer and Lindenmayer 2007). The ability of scientists and conservation practitioners to reliably assess land‐use impacts on biodiversity relies on access to spatially consistent information on anthropogenic use throughout the area of interest.

Depending on the spatial extent and needs of a study, land‐use data can be derived from three sources: direct surveys, remote sensing, or model‐based analyses. Each approach presents benefits and limitations to their use. Direct surveys are generally performed across relatively local extents for specific purposes where they can provide reliable fine‐grained information. However, the time and cost of implementing such surveys limits their utility for large‐scale assessments.

Classification of spectral data from remote sensing (e.g., MODIS satellite imagery) is used to map the distribution of land‐cover classes (De Fries et al. 1998; Hansen et al. 2000; Friedl et al. 2002; Chen et al. 2015). While classified spectral data produce high‐resolution snapshots of the current state of land cover, these analyses may not differentiate between anthropogenically modified ecosystems and spectrally similar natural ecosystems (Hurtt et al. 2001; Kerr and Ostrovsky 2003; Haines‐Young 2009). For example, a remotely sensed grassland classification could include undisturbed natural grassland or grassland created by clearing natural forest for domestic livestock grazing. Land‐use classifications in this example are markedly different – primary and pasture, respectively, and the capacity for local biodiversity retention is likely to differ greatly between such areas (Zimmermann et al. 2010).

While land cover delineates differences in the physical cover of the Earth's surface, land‐use classifications describe the type and extent of human influence (Fisher et al. 2005). Spatial distribution in land use is typically described using multiscale modeling techniques that integrate multiple local, regional, and global influential drivers of land‐use change (Veldkamp and Lambin 2001; Verburg et al. 2004; Verburg and Overmars 2009). These models classify land use, land‐use intensity, and combinations of land‐use and land‐cover classes (Verburg et al. 2002; Verburg and Overmars 2009; Connor et al. 2015).

At their simplest, land‐use models generate spatial predictions of land‐use classes by combining commodity‐based economic models with information describing land productive capacity (Heistermann et al. 2006). Present‐day land‐use data at a variety of spatial scales (e.g., country, regional and/or subregional statistics) are used to initialize the model (Heistermann et al. 2006). Models then balance the trade‐offs between different land uses and spatially allocate predictions of land‐use type. This is achieved by maximizing economic benefit to meet present‐day production levels (reported by country, regional, or subregional statistics) or future production targets (Verburg et al. 2004). Land‐use models can then provide spatial predictions of both present‐day land use and projections of future land‐use change following multiple scenarios of future human development and growth (Verburg et al. 2008; van Delden et al. 2010).

Increased technical and computational complexities have limited the production of a global fine‐grained land‐use model (Heistermann et al. 2006). Rather, land‐use modeling scientists have focused on producing fine‐grained regional models (Verburg et al. 2002; Sohl et al. 2012; Connor et al. 2015). There exist a number of models that classify land‐use globally; however, these are much coarser grained (≥10 km^2^) than regional models (Erb et al. 2007; Havlík et al. 2011; Hurtt et al. 2011a; Letourneau et al. 2012; Souty et al. 2012).

Land‐use classifications contain substantial information of relevance to biodiversity research and conservation assessment (Newbold et al. 2015). By describing anthropogenic influence in an area, these classifications reduce the potential for geographically variable interpretations of biodiversity outcomes, thereby providing spatially equivalent measures for large‐scale biodiversity analyses. However, many ecological processes affected by land use operate at a much finer spatial grain than that provided by current global land‐use models (Pereira et al. 2010). Refining the spatial resolution of global land‐use modeling and mapping could help to better account for relevant ecological processes in assessing impacts of land‐use change on biodiversity and to better integrate consideration of these impacts across local, regional, and global scales.

We here explore the use of statistical downscaling (Atkinson 2013) as a relatively straightforward and cost‐effective means of enhancing the spatial resolution of global land‐use modeling. This involves fitting a statistical model relating coarse‐scaled spatial patterns in the distribution of land‐use classes to finer‐scaled land cover, climate, landform, and anthropogenic influence layers, and then using this fitted model to map land use at the finer spatial resolution. We describe a new technique for accommodating multiclass proportional data in statistical downscaling and apply this to the 0.5° Land‐use Harmonization dataset of Hurtt et al. (2011b) to produce a global, 30″ (approx. 1 km^2^) dataset of five land‐use classes (primary habitat, secondary habitat, cropland, pasture, and urban). The resulting dataset represents a critical step toward integrating land use into global biodiversity assessment at a spatial resolution of greater ecological relevance than has been possible to date.

## Methods

### Inputs

The Land‐use Harmonization dataset (LUH; Hurtt et al. 2011b) is a global time series of past, present, and future land use at 0.5°, spanning 1500–2100. The dataset describes the proportional cover in each 0.5° grid cell of five land‐use categories: primary habitat, secondary habitat, cropping, pasture, and urban (see Table 1 for additional descriptions). We selected this dataset because of its global extent and wide temporal coverage and to ensure compatibility with recent work establishing a global database of the response of biodiversity to land‐use pressures (see Hudson et al. 2014). In this first application of our downscaling method, we downscaled a time point representative of the present day (2005) to produce fine‐grained, global estimates of the five land‐use classes. However, we have plans to extend these methods to produce fine‐grained projections of future land‐use change, which are temporally and spatially consistent.

**Table 1:** Descriptions of the five Land‐use Harmonization land‐use classes that were used in our downscaling model

Land‐use type	Description
Primary	Undisturbed natural habitat
Secondary	Recovering, previously disturbed natural habitat
Cropland	Land used for crop production (e.g., wheat, rice, corn)
Pasture	Land used for the grazing of livestock
Urban	Land converted to dense urban settlement

To downscale the LUH dataset, we required fine‐grained covariate layers that were identified a priori as potentially correlating with fine‐grained land‐use patterns. There are instances where relying solely on satellite‐based information can lead to a misinterpretation of relationships (Gilmore 2015). To mitigate this, we selected satellite derived and additional data sources as covariate data. We accessed best‐available spatial data on climate, landform, soil, land cover, human population density, and accessibility and derived 30″ layers of each. Fine‐grained cells were defined as useable land, water, or ice, using a land mask consistent with the WorldClim dataset (Hijmans et al. 2005) (See Table 2 for further description of input data sources).

**Table 2:** Description of data layers used as covariates or masking layers during the downscaling process

	Description	Units	Use	Source
Climate				
EARS	MOD16 dataset gap filled with Annual Actual Evaporations calculated as the sum of monthly EA derived using the Budkyo framework based on WorldClim climatic data, using PAWHC calculated from 1 km Soil Depth from www.soilgrids.org combined with AWC from the Harmonized World Soil Database	mm	Predictor	Hijmans et al. (2005); Mu et al. (2007); FAO/IIASA/ISRIC/ISSCAS/JRC (2012); Hengl et al. (2014)
MAT	Mean Annual Temperature with maximum and minimum temperature corrected for radiation differences due to variation in terrain based on Danielson and Dean (2011) following Wilson and Gallant (2000)	°C	Predictor	Wilson and Gallant (2000); Hijmans et al. (2005); Danielson and Dean (2011)
PTA	Annual precipitation. Sum of monthly precipitation from WorldClim	mm	Predictor	Hijmans et al. (2005)
TWI	Topographic Wetness Index. Calculated at 9″ and upscaled to 30″		Predictor	Reuter and Hengl (2012)
Landform/substrate				
ICE	Presence of permanent ice	Binary	Mask	Olson et al. (2001)
SLP	Slope calculated at 9″ and upscaled to 30″	%	Predictor	Reuter and Hengl (2012)
SOC	Soil Organic Carbon content. Weighted average of all depth classes	g/kg	Predictor	Hengl et al. (2014)
WATER	Presence of permanent water bodies	Binary	Mask	Lehner and Doll (2004)
Anthropogenic				
ACC	Global Accessibility Index. The travel time to the nearest population center of 50,000 or more		Predictor	Uchida and Nelson (2010)
POP	Population density.	People/km^2^	Predictor	Balk et al. (2005)
Land cover				
CLC	Consensus land cover. 30″ land‐cover product made by harmonizing multiple products	%	Predictor	Tuanmu and Jetz (2014)

PAWHC is the Plant Available Water Holding Capacity of soil. AWC is the Available Water Capacity of soil. A variable used as a predictor describes a fine‐grained covariate used in the regression model. A variable described as a mask describes a binary (0 or 1) variable used to determine whether values from a cell are included in the model or excluded.

The consensus land‐cover dataset (Tuanmu and Jetz 2014), that was used as input to the downscaling (see Table 2), describes 12 land‐cover classes. Due to strong collinearity between some classes, the consensus land‐cover data were rotated using a principal components analysis to produce the minimum uncorrelated components accounting for 99% of the variation within the 12 classes. The resulting principal components were used as predictor variables in the downscaling procedure, rather than raw land‐cover data.

### Downscaling algorithm

Statistical downscaling uses statistical methods to translate relationships between coarse‐grained response data and (multiple) fine‐grained covariate data into fine‐grained predictions of the response (Atkinson 2013). These methods usually rely on regression modeling to identify correlative relationships between the response variable and the covariates (Dendoncker et al. 2006; Atkinson 2013; Poggio and Gimona 2013). Applying these methods to the global LUH dataset required an approach capable of describing the complex spatial structure within a constrained dataset.

The LUH dataset has five classes to downscale, with each representing a fraction of coarse‐grained land area occupied by the respective land‐use class. Relationships between this dataset and our chosen fine‐grained covariates will be complex and nonlinear. Thus, our method must accommodate nonlinear patterns and the fine‐grained predictions should, when aggregated back to 0.5°, approximate the original coarse‐grained LUH predictions. Our fine‐grained data should also represent the fraction of each land use within a fine‐grained grid cell *j*, so predictions of all five land uses must obey the constraints

(1) $$\sum_{a=1}^{5}lu_{j,a}=1,$$

(2) $$0\;\leq \;lu_{j,a}\;\leq \;1.$$

Here, *lu*_*j*,*a*_ is the proportion of land use at a cell and *a* represents one of the five classes being downscaled. A multinomial logistic regression could meet these constraints (e.g., Dendoncker et al. 2006). However, this would limit our ability to fit complex nonlinear patterns expected in this dataset.

**Figure 1:**
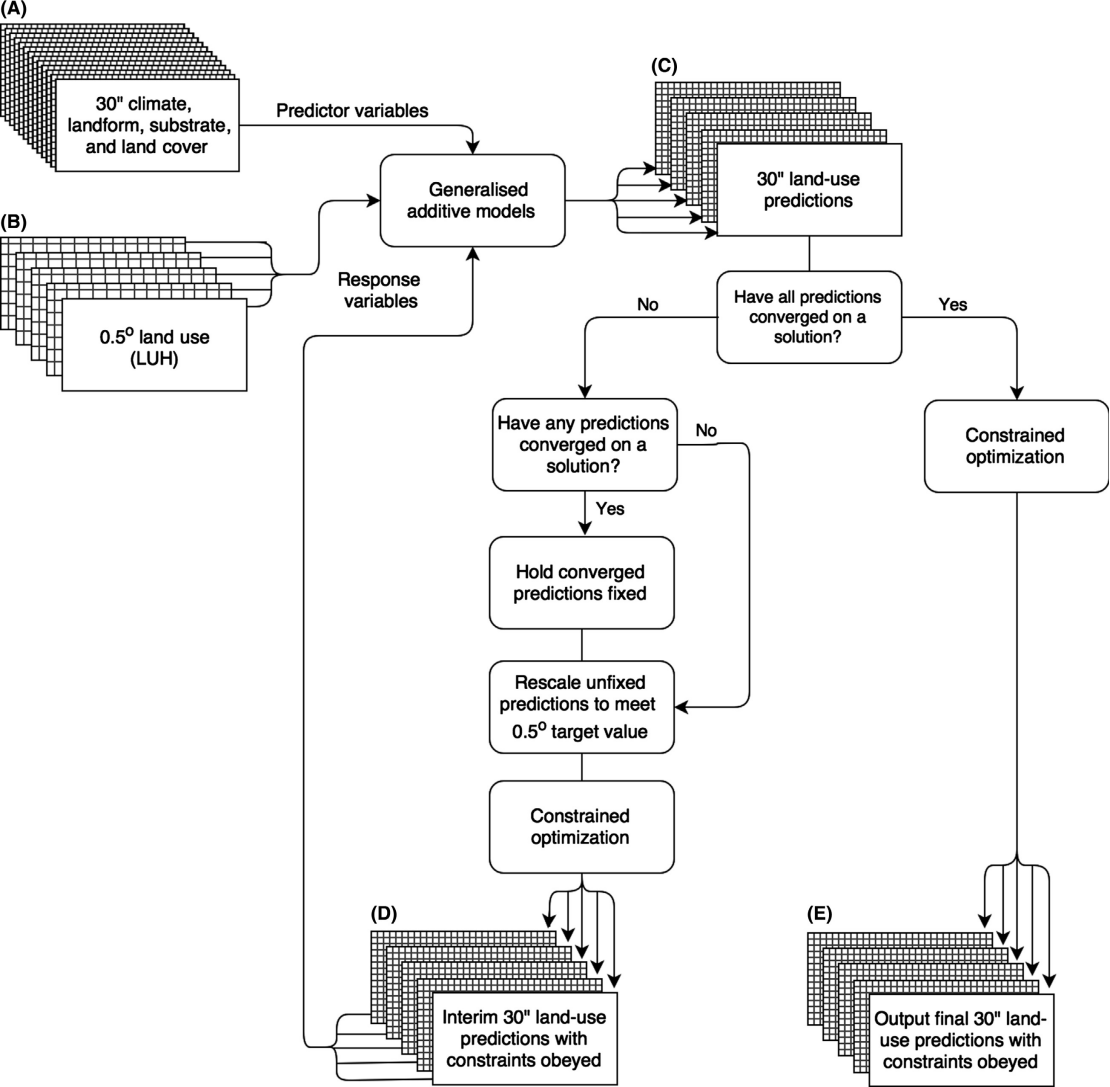
A schematic of the algorithm used to downscale the Land‐use Harmonization dataset (2005) into fine‐grained (30″) land use.

The new method to statistically downscale proportional land‐use data obeying the constraints in equations (1) and (2) is shown in Figure 1. We extended the method of Malone et al. (2012), using a combination of nonlinear regression and constrained optimization. This produced multiple high‐resolution layers based on the global LUH dataset (Hurtt et al. 2011b). We now discuss our approach in detail.

To account for the nonlinear land‐use patterns, we use a distinct generalized additive model (GAM) for each land‐use class. These used a quasi‐binomial error family with logistic link function to account for the 0–1 bounded constraint (eq. (2)). We set coarse‐scale land‐use values from the LUH layers as initial response variables for five separate GAMs (Fig. 1A). These were modeled against the fine‐grained climatic, land‐form, and land‐cover variables (described in the “inputs” section above) to predict each land‐use at 30″ (Fig. 1B,C).

With the five GAMs constructed, we then calculated the 0.5° grid cell average for these predictions and compared them to the original LUH values. The predicted 30″ land‐use values were subsequently rescaled multiplicatively for each land‐use class *a* following the expression

(3) $$lup_{j,a}=lu_{j,a}\cdot \left(\frac{LU_{i,a}}{\sum_{j=1}^{N_{i}}lu_{j,a}/N_{i}}\right),$$

where *lu*_*j,a*_ is the GAM predicted land‐use *a* in fine‐grained cell *j*,* LU*_*i,a*_ is the proportion of land‐use *a* in coarse‐grained cell *i,* and *N*_*i*_ is the number of available fine resolution cells within each coarse‐resolution cell *i*. This produces a scaling adjusted prediction *lup*_*j,a*_ for each land‐use *a* at the fine‐grained cell *j*.

Given that each land‐use prediction is based on separate GAMs, the predictions may not obey the constraint in equation (1). Additionally, the rescaling in equation (3) may cause predictions to violate the constraint in equation (2). To balance our predictions while obeying all constraints, we passed the rescaled *lup* predictions of all five land‐uses and their associated standard errors (*σ*) (derived from the GAMs) through a constrained optimization algorithm. This algorithm identifies, per fine‐grained cell *j*, the optimal configuration of the five classes by minimizing the χ^2^ function $\left(\chi^{2}=\sum_{a=1}^{5}{\left(luc_{j,a}-lup_{j,a}\right)}^{2}/\sigma_{a}^{2}\right)$ , where *luc*_*j*,*a*_ is land use from the constrained optimization. Accounting for the standard error allows the algorithm to preferentially adjust uncertain GAM predictions, while maintaining both constraints.

The interim constrained estimates of all five land uses are then passed back into the GAMs as response variables for use with the fine‐grained predictors in the next iteration (Fig. 1D). This is repeated until the average land‐use interiteration prediction difference at 30″ is less than 0.001. When predictions for one land‐use converge, that class is fixed and the procedure repeats until all classes converge. At this point, the optimal GAM solution is assumed to be found. The predictions are passed to the constrained optimization algorithm a final time, ensuring all constraints are obeyed and the output is returned (Fig. 1E).

### Global implementation of the downscaling algorithm

It was assumed that the form of the relationship between each land‐use class and the predictor variables might vary across different regions due to local differences in land‐use conversion patterns. These differences could relate to both environmental and anthropogenic factors (Lambin et al. 2003). Consequently, downscaling the land‐use data in a single global model would have been inappropriate. Instead, we partitioned the global terrestrial region into 61 bio‐realms (Lee and Jetz 2008), representing unique combinations of biome and biogeographical realm, based on the WWF ecoregional classification (Olson et al. 2001) and downscaled each bio‐realm separately.

The lower level division of realms and biomes into ecoregions, as described by Olson et al. (2001), was not used because our downscaling method required a sufficient spatial coverage of coarse‐grained land‐use information to establish relationships between land use and fine‐scaled covariates within each model. It was felt that ecoregions were generally too small for this purpose, whereas bio‐realms were both sufficiently large to provide adequate sample sizes for downscaling, while allowing for any major differences in relationships between covariates and land use as a function of environmental and anthropogenic differences between these units.

In reality, bio‐realm boundaries are not sharp divisions and land‐use patterns may be similar in neighboring regions. Thus, in addition to all coarse‐grained cells where a fine‐grained cell belonged to the target bio‐realm, we included a buffer of one 0.5° cell from neighboring bio‐realms, allowing within bio‐realm land‐use patterns to be influenced by neighboring bio‐realms.

The target LUH proportions are only for available land in each grid cell. Consequently, where a cell contains hydrological features (lakes, rivers, and ocean) or permanent ice, the target proportions were adjusted to represent the remaining fraction of land in the cell. Fine‐grained cells designated as water or ice were then excluded from subsequent analyses.

The final downscaled, 30″ land use from the 61 areas was converted into five global mosaics (one per land‐use class). Each area was joined to adjacent areas by removing buffering cells from neighboring regions. We treated each model output as the best solution for the bio‐realm being modeled and, as such, made no attempt to smooth across bio‐realm boundaries. Rather, we show the consistency of predictions between adjoining models in areas where predictions overlapped (see description in validation section below).

### Validation

We compared the downscaled data with the original LUH dataset by aggregating the fine‐grained layers to their original coarse grain. We fitted linear models to the aggregated output of each downscaling model, evaluating differences by comparing goodness of fit (*R* ^2^). The downscaled data and LUH data were also aggregated into global and regional (realm) proportions of land use and differences between the two populations compared. A χ^2^ goodness‐of‐fit statistic was not calculated as the extremely large sample sizes would result in statistically significant results even when the actual changes in proportions were minor.

Consistency between model predictions was assessed by extracting the “buffer” cells from models and comparing overlapping model predictions. Compositional differences between predicted land uses were calculated using Bray–Curtis dissimilarity for each cell (Bray and Curtis 1957). The spatial configuration of mean dissimilarity and the absolute differences in prediction for individual land uses in overlapping regions are presented here.

There are few datasets available to evaluate the accuracy of our fine‐grained predictions. Most datasets are restricted in spatial extent or lack statistical independence because they have been used in our input layers. For example, the LADA land‐use systems maps (Nachtergaele and Pertri 2011) have been generated using the GLC2000 land‐cover maps (Bartholomé and Belward 2005) and the GRUMP population density maps (Balk et al. 2005), both of which have been used as predictor variables during our modeling. Conversely, other existing spatial datasets have been derived from data that are also used in generating our response variables (the coarse LUH data). For example, the ANTHROME land‐use maps (Ellis et al. 2010) were derived from the HYDE models (Kees Klein et al. 2011) which are also a major component of the LUH models. Nonetheless, we used two available independent global datasets to critically evaluate our methods.

The Projecting Responses of Ecological Diversity In Changing Terrestrial Systems (PREDICTS) project has established an extensive global dataset for assessing impacts of land use on biodiversity, by collating both biological and land‐use information for a large number of sites extracted from previous studies (Hudson et al. 2014). We used the spatial location and land‐use classifications from PREDICTS sites surveyed in 2004–2006 (1 year either side of the downscaled land use) to validate our downscaled predictions (accessed 28 July 2015).

The PREDICTS database describes land use at each site categorically for multiple patch sizes. Our downscaled layers predict the proportion of each land use within a 30″ grid cell; thus, a direct 1:1 comparison between datasets is not possible. Instead, we extracted predicted land use for the PREDICTS sites from the downscaled layers and from the original coarse‐grained LUH dataset. We tested the ability of predictions to discriminate between each of the five land uses in the PREDICTS database (treated as five separate binary indicators). This was achieved by calculating the relative operating characteristic (ROC) curves for land‐use predictions from the LUH and downscaled datasets (Pearce and Ferrier 2000). The area under the ROC curve (AUC) was estimated using the Mann–Whitney statistic, and significant differences in AUC were tested using confidence intervals calculated by the bootstrap method detailed in Carpenter and Bithell (2000) via the R package pROC (v 1.8; Robin et al. 2011).

Additional validation was carried out on the cropping layer using the remotely sensed geoWiki cropping dataset (Fritz et al. 2015). This global dataset contains proportional cropping values at 30″ for 2005, making it ideal for comparison with our downscaled cropping data. The geoWiki cropping layer was compared to the downscaled and LUH cropping layers using two linear models (geoWiki vs. downscaled cropping and geoWiki vs. LUH cropping). Each model's intercept was fixed to zero, to ensure we assessed a direct 1:1 relationship between the response and predictor data. We assessed the difference in estimated slope and *R* ^2^ for the two models when regressed against the geoWiki data.

## Results

**Figure 2:**
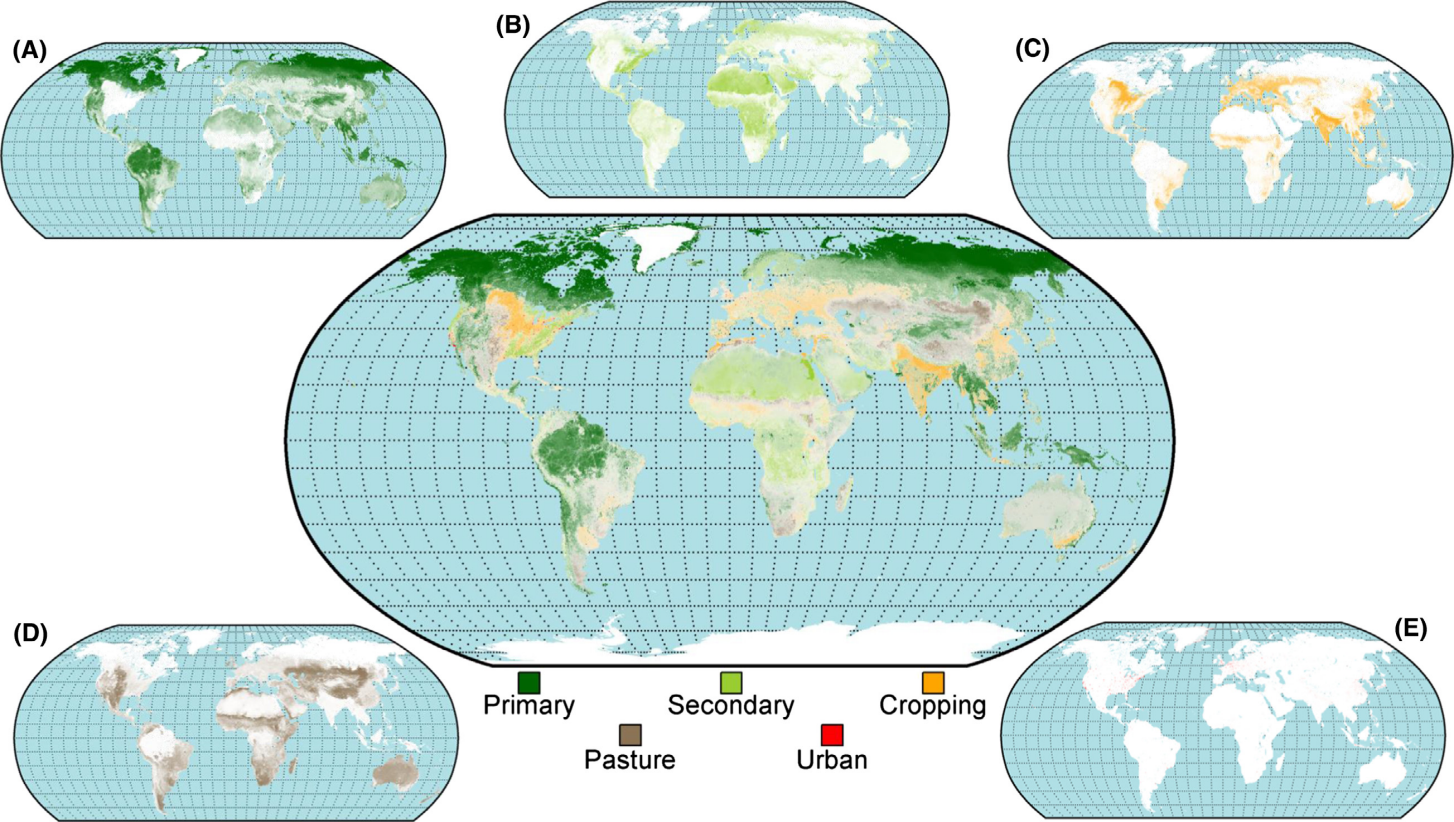
Map showing the global results of the downscaling analysis. The transparency of individual colors (representing each land‐use class) has been varied according to the proportion of that class predicted to occur within a given cell. Cells with a single dominant land use will show a color closest to the color representing that land‐use class, whereas cells predicted to contain a mixture of classes will exhibit a mixed color. Inset panels a–e show the spatial distribution of each land use individually where (A) is primary, (B) is secondary, (C) is cropping, (D) is pasture, and (E) is urban. See Figures S1–S5 for high‐resolution global maps showing each land‐use individually.

**Figure 3:**
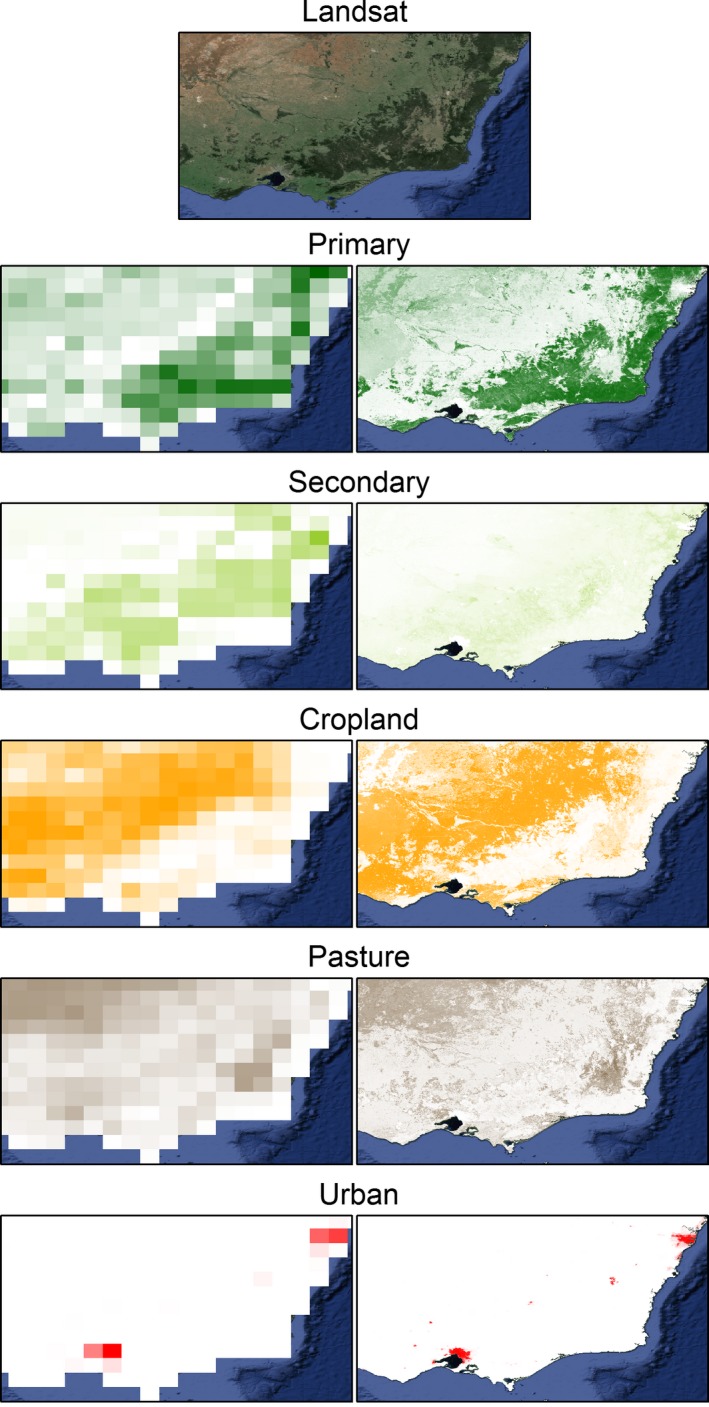
Visual comparison of the south‐east Australian region with true color landsat imagery and the proportions of each of five land‐use classes predicted to occur by the original coarse‐grained (0.5°) Land‐use Harmonization dataset (left panel) and the fine‐grained (30″) downscaled land‐use datasets (right panel). Color intensity in panels in rows 2–5 represent low to high proportions of land use within a pixel.

We combined our best estimate results of the downscaling models for each of the 61 bio‐realms to produce global, spatially complete, 30″ land‐use estimates for all five land‐use classes (Fig. 2. See Figs S1–S5 for maps showing individual land‐uses). All estimates obeyed the constraints given in equations (1) and (2). The downscaled layers exhibited far finer spatial patterns when compared with the original datasets (e.g., Figs 3 and S6–S8 for additional examples).

**Figure 4:**
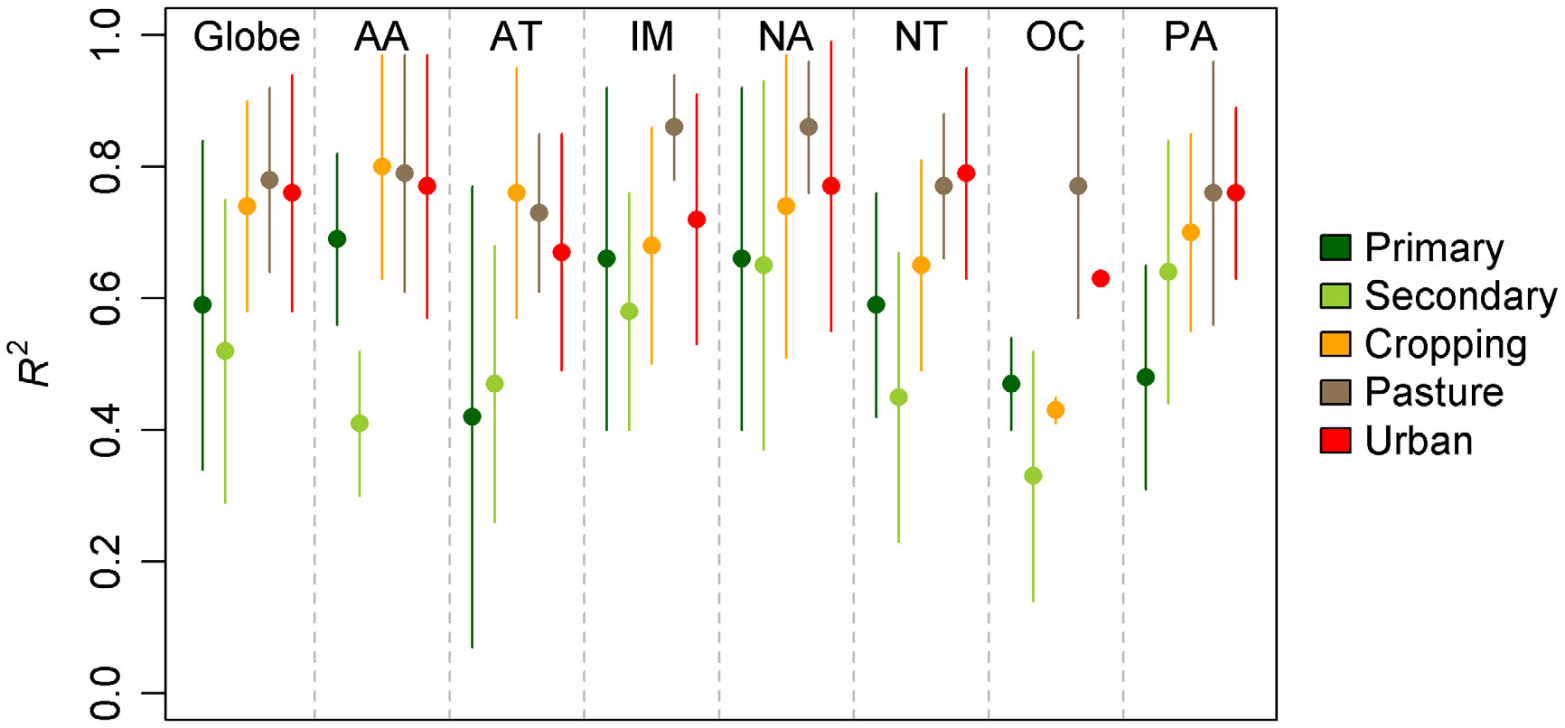
Comparison of *R* ^2^ values obtained from regressing the proportion of each of five land‐use classes predicted to occur within a 0.5° cell from the downscaled 30″ datasets and the original coarse‐grained (0.5°) Land‐use Harmonization datasets. Values are shown as mean ± standard deviation. Realms: AA: Australasia, AT: Afrotropics, IM: Indo‐Malaysia, NA: Nearctic, OC: Oceania, PA: Palearctic.

**Figure 5:**
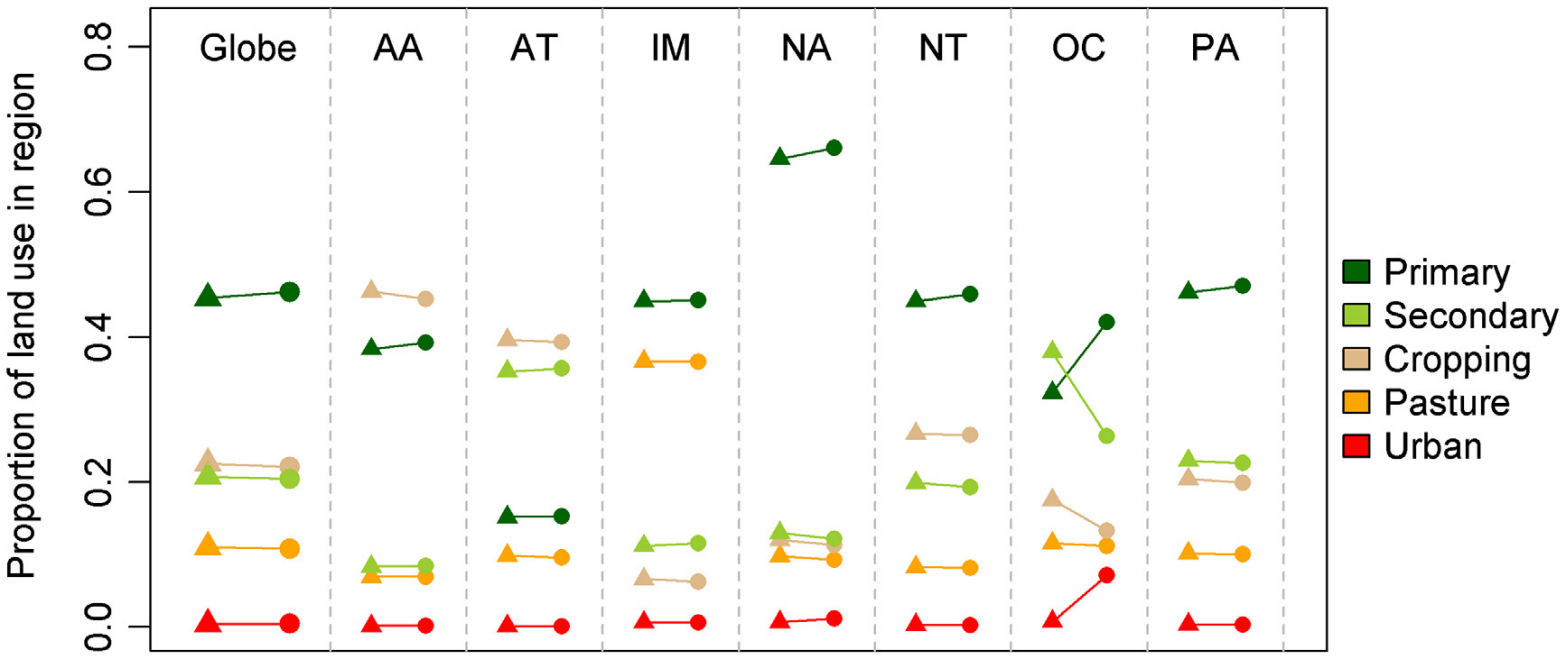
Comparison of the aggregated proportions of each of five different land‐use classes from both the Land‐use Harmonization dataset (triangles) and the results of the downscaling procedure (circles) aggregated globally and for each realm. AA: Australasia, AT: Afrotropics, IM: Indo‐Malaysia, NA: Nearctic, OC: Oceania, PA: Palearctic.

Models predicted similar proportions of land use within the coarse‐grained cells compared to the original LUH values (Global mean *R* ^2^ ± SD: 0.68 ± 0.19, Fig. 4 and Table S1). Globally, the dataset estimated the proportions of all land between 60°S and 90°N to contain 46.2% primary habitat, 20.4% secondary habitat, 10.8% cropland, 22.1% pastoral land, and 0.5% urban areas. Aggregated values compared well with the original LUH estimates at both global and realm level (Fig. 5; Table S2). An exception is the Oceania realm where our models predicted greater proportions of primary and urban land use and less secondary and pasture land use compared with the LUH dataset (Fig. 5; Table S2).

**Figure 6:**
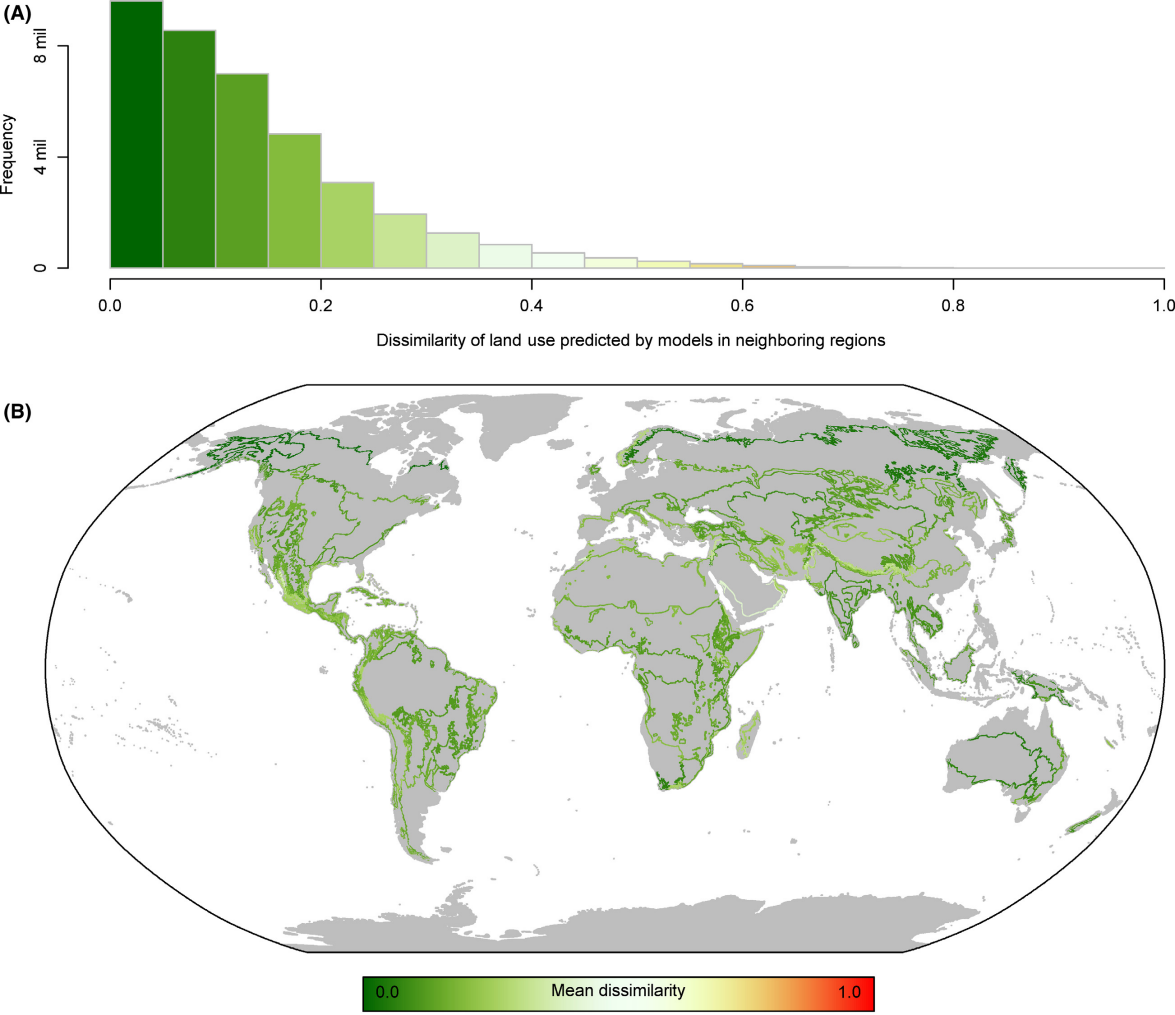
Distribution of Bray–Curtis dissimilarity values calculated in the areas where two neighboring models provided overlapping predictions. (A) The frequency of values from the global sample of overlapping cells. (B) The spatial arrangement of dissimilarity values, colors represent the mean dissimilarity of all cells within each unique boundary between two unique pairs of bio‐realm.

Overall, overlapping predictions between neighboring bio‐realm models were reasonably consistent with dissimilarity values, averaging 0.13 ± 0.11 for the globe and for individual realms 0.08 ± 0.07 (Australasia), 0.16 ± 0.11 (Afrotropics), 0.13 ± 0.10 (Indo‐Malaysia), 0.10 ± 0.11 (Nearctic), 0.16 ± 0.10 (Neotropics), and 0.12 ± 0.12 (Palearctic) (Fig. 6A). However, several areas showed buffer zone downscaling predictions with higher dissimilarities compared to neighboring models (Fig. 6B), suggesting differences in underlying GAM response functions between these models. The greatest mismatches occurred in the Arabian Peninsula where boundaries had average dissimilarity values ranging 0.41–0.59 (Fig. 6B). Comparisons of the absolute difference between each land‐use class for overlapping regions highlighted that discrepancies detected through calculating dissimilarity values were primarily caused by variation in primary and secondary habitat predictions between bio‐realms that were topographically complex (Fig. S9).

### Validation with independent datasets

The PREDICTS database contained a total of 3669 unique sites for which land use was recorded within the period of interest to our analysis (2004–2006). Of these, the number of sites available per land‐use class was 1302 for primary, 1112 for secondary, 555 for cropping, 564 for pasture, and 136 for urban. Sites were spread globally with an average of 522 ± 450 sites per realm (min: 3 for Oceania. max: 1191 for Neotropics). However, a bias existed toward sites occurring in the Western Palearctic and Neotropics (23% and 32% of all sites, respectively).

**Figure 7:**
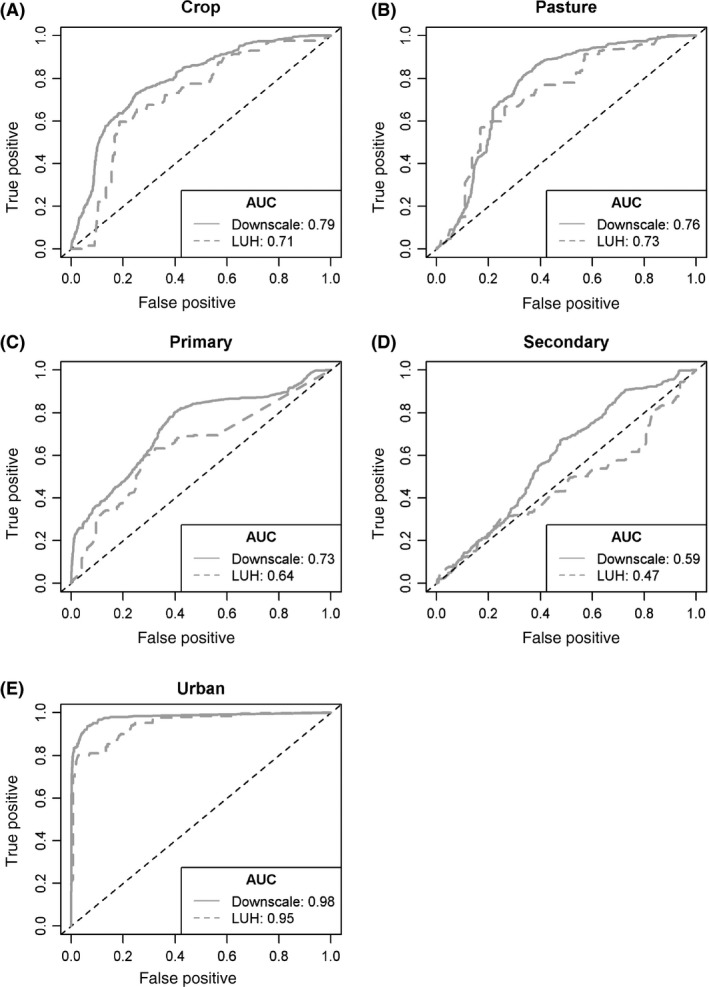
Relative operating characteristic curves for comparisons of the PREDICTS validation dataset and each of the five different land‐use classes for the downscaled data (solid gray line) and the Land‐Use Harmonization dataset (dashed gray line). (A) Cropping land‐use. (B) Pasture land‐use. (C) Primary habitat. (D) Secondary Habitat. (E) Urban land‐use.

Our downscaled datasets achieved a significant improvement in AUC compared with the LUH datasets at PREDICTS sites for all five land uses (*P* < 0.0001 in all cases; Table S3). The AUC value increased between 0.03 and 0.12 depending on the land‐use class. Discrimination was good to very good (AUC > 0.7) for all downscaled predictions (AUC range 0.73–0.98; Fig. 7) with the exception of secondary habitat which showed poor discrimination between PREDICTS sites classed as secondary (AUC = 0.59).

Our downscaled cropping data compared well with the geoWiki cropping dataset. There was an improvement in cropping estimates relative to the original coarse‐grained LUH data. The two linear models showed an improvement in the *R* ^2^ of 0.12 and an increase in the slope coefficient of 0.04 between the two models (*R* ^2^ 0.76 and 0.64 and slope coefficients 0.96 and 0.92 for downscaled and LUH models, respectively).

## Discussion

Using our statistical downscaling method, we have been able to derive a set of globally complete and consistent fine‐grained land‐use layers for the present day (2005). These layers estimate the distribution of land‐use classes at a spatial grain more relevant to local‐scale ecological processes and land management practices affecting the distribution and state of species and biological communities across the landscape. Throughout this study, we have described our approach as downscaling coarse‐grained land‐use data using fine‐grained covariates, including remotely sensed land cover. Some readers might, however, find it useful to view the approach from an alternative perspective. This involves thinking of fine‐grained land cover as the primary source of data and viewing our modeling as translating this layer into fine‐grained land use, based on observed correlations with coarse‐grained land‐use, and information on other fine‐grained environmental covariates.

Our downscaled land‐use data compared well with the original 0.5° LUH dataset, and the independent PREDICTS and geoWiki validation data (Hudson et al. 2014; Fritz et al. 2015). Our dataset estimated 32.77% of terrestrial habitat in 2005 was under some level of human use (10. 8% cropping, 22.1% pasture, and 0.5% urban). Previous studies covering a similar time period (2000–2002) estimate between 11.8% and 12% of global terrestrial land were used for cropping; 22–26% for pasture and 0.24–0.51% for urban use (Bartholomé and Belward 2005; Potere and Schneider 2007; Ramankutty et al. 2008; FAOSTAT 2013). It should be stressed that our analyses are guided and constrained by the original coarse‐grained LUH dataset from which broad‐scaled spatial patterns of land use are derived. As such, validation against other land‐use data needs to be viewed within the constraints of the original coarse‐grained LUH dataset. The method presented here is, however, relatively generic and could be applied to other land‐use or land‐cover datasets. For example, by applying this method to the GLOBIO/IMAGE 10 km^2^ resolution datasets, fine‐grained layers spanning the numerous classes contained within that dataset could be derived (Letourneau et al. 2012). Similarly, replacing the consensus land‐cover (Tuanmu and Jetz 2014) dataset, as the source of land‐cover covariates employed in our initial analysis, with a land‐cover dataset with multiple time points (e.g., the MODIS land‐cover data; Friedl et al. 2002) could enable the generation of a time series of downscaled recent land‐use change.

One realm, Oceania, showed higher differences in the proportions of land uses predicted between the new downscaled layer and the original LUH dataset (Fig. 5). This is the smallest realm by landmass, consisting of many islands scattered throughout the Pacific Ocean. There are 288 coarse‐grained grid cells in Oceania within the LUH dataset, representing 57,386 fine‐grained cells in our downscaled layers. The power of our technique comes from its ability to identify covariate relationships from a large number of coarse‐grained grid cells and translate these into fine‐grained predictions. The small number of coarse‐grained cells, sparsely distributed across a large region, likely contributed to the discrepancies between datasets.

The buffering approach we employed allows us to express uncertainty in our estimates at the boundaries of neighboring models and identify areas where predictions differ (Fig. 6 and Fig. S9). Boundary effects were strongest in areas where topographically complex bio‐realms met more uniform bio‐realm (i.e., mountainous regions meeting plains; Fig. 6 and Fig. S9). In these areas, models describing mountainous regions have probably fitted relationships to much steeper gradients in topography and climate compared with models of areas where topography was more uniform. Thus, it is understandable that predictions in areas novel to the model (i.e., a mountains model predicting into a plains region or vice versa) may differ, creating a disjunct at these boundaries.

Boundaries could be minimized by spatially smoothing bio‐realm edges (e.g., using inverse distance weighting or smoothing splines) but while this would be visually satisfying, the underlying disjunct in model outputs would still exist. We partitioned the modeling using the global classification of bio‐realm provided by Olson et al. (2001), who delineated these regions using broad differences in landform, climate, and vegetation cover. An alternative approach might be to develop anthropocentric divisions that discriminate major spatial or cultural differences in land utilization (e.g., Ellis et al. 2010). However, such a global classification as large‐scale contiguous spatial blocks has yet to be developed.

Recently, Newbold et al. (2015) presented the first global‐scale assessment of local land‐use effects on biodiversity. Their study used the PREDICTS database, giving their analyses unprecedented spatial and taxonomic coverage. The authors made use of the best‐available spatial data on land use when projecting their results to areas beyond their study sites. However, those data were of a coarse spatial grain (0.5°) which may have masked some of the local ecological processes the study was seeking to quantify. The integration of the PREDICTS dataset with the global fine‐grained land‐use layers presented here would allow analyses employing the methods of Newbold et al. (2015) to report on biodiversity at a finer spatial grain than previously possible.

While our downscaling of global land use was purposely designed to complement the data and analyses arising from the PREDICTS project, the product we have generated has potential for broader application in biodiversity and conservation studies. For example, the translation of land‐cover mapping into anthropogenic land‐use types, achieved by our downscaling approach, opens up opportunities for a fine‐grained analysis of the global state of natural habitat within and outside protected areas (e.g., Hoekstra et al. 2005). These downscaled data could also be used to generate an index of habitat condition and, using community level modeling techniques (such as generalized dissimilarity modeling; Ferrier et al. 2007), global scale analyses assessing the state of regional biodiversity could be undertaken (e.g., Allnutt et al. 2008). Additionally, these data could feed into conservation prioritization analyses addressing spatial relationships between anthropogenic land‐use change and needs for biodiversity protection (e.g., Moilanen et al. 2011).

We have described here the downscaling of present‐day (circa 2005) data but have not extended this to produce fine‐grained future land‐use projections. One of the ultimate goals of our research is to utilize the coarse‐grained predictions of land use in the LUH dataset, coupled with the fine‐grained present‐day predictions presented here to produce spatially and temporally consistent fine‐grained projections of future land use. Currently, however, the method leverages much of its spatial pattern through present‐day remotely sensed land‐cover maps. The lack of future land‐cover predictions means our method cannot be simply used to produce fine‐grained future scenarios of land use. This problem could be partially overcome by replacing the land‐cover covariates by the land‐use outputs produced by downscaling present‐day land use and then employing these alongside abiotic covariates in downscaling coarse‐scaled projections of future land use. However, the production of the 2005 layers relied on accessing 1024 GB of RAM, spread between 17 compute nodes, run for almost a month. To repeat this process iteratively for numerous future time steps would require major improvements in computational power or efficiency to be completed within a reasonable timeframe. However, refinements and extensions of the current method are being actively pursued that will allow us to build on the datasets presented here to produce a projected time series of fine‐grained future and past land use.

One component of land use that is currently missing from our downscaled maps is the intensity of anthropogenic use. The intensity of a particular land use can have major effects on the outcomes for local biodiversity (Klein et al. 2002; Kleijn et al. 2009; Newbold et al. 2013; Tuck et al. 2014; Kehoe et al. 2015) and the ability to divide our maps into different intensities would be a major improvement. Defining and mapping land‐use intensity of multiple land‐use classes, particularly at global extents, is challenging (Kuemmerle et al. 2013) and doing this well will require methodological advances on several fronts. These include analytical improvements (e.g., improved use of remote sensing data) as well as greatly improved primary data with large spatial coverage for all aspects of land‐use intensity, including inputs (e.g., fertilizer type), outputs (e.g., yields, felling ratios), and unintended outcomes (e.g., net affect to biodiversity) (Erb et al. 2013). However, this is an active field of research (Jain et al. 2013; Václavík et al. 2013; Erb et al. 2014; Petz et al. 2014; Rufin et al. 2015) and continuing advances may make it possible to combine our maps with additional data to develop fine‐grained maps of land‐use intensity in the near future.

For example, Newbold et al. (2015) inferred intensity of secondary land uses from the LUH dataset by substituting time since conversion for intensity, allowing them to make the distinction between young, intermediate, and mature secondary habitat. A similar method could be incorporated into our downscaling approach with the addition of past time steps. Additionally, because our estimates are continuous and not categorical, an inference of intensity could be made based on a simple “rule of thumb” being applied – for example, based on the estimated proportion of a grid cell belonging to a given land‐use class (LU): 0 < LU < 0.33 = low intensity, 0.33 < LU < 0.66 = moderate intensity and 0.66 < LU < 1 = high intensity. However, this would address only a subset of the complex multidimensional, and spatially variable, factors relating to land‐use intensity (Erb et al. 2013).

Our new approach to the constrained downscaling of land‐use data successfully reproduces the original coarse‐grained data and results in qualitatively sensible fine scale outputs. Quantitative analyses of aspects of the outputs confirm the downscaling reduces spatial error when compared to the coarse‐grained inputs. While applied to land‐use data in this worked example, the approach has wider potential application downscaling coarse‐scaled mapping of other environmental variables.

## Data Accessibility

All five globally complete land‐use data layers are available via the CSIRO's Data Access Portal which can be accessed via the link: http://doi.org/10.4225/08/56DCD9249B224.

## Conflict of Interest

None declared.

## Supporting information


**Figure S1.** Global distribution of primary habitat predicted to occur at 30 arc sec resolution produced by downscaling the coarse grained (0.5°) Land‐use Harmonisation dataset. Colours are ramped light (low) to dark (high).
**Figure S2.** Global distribution of secondary habitat predicted to occur at 30 arc sec resolution produced by downscaling the coarse grained (0.5°) Land‐use Harmonisation dataset. Colours are ramped light (low) to dark (high).
**Figure S3.** Global distribution of cropland predicted to occur at 30 arc sec resolution produced by downscaling the coarse grained (0.5°) Land‐use Harmonisation dataset. Colours are ramped light (low) to dark (high).
**Figure S4.** Global distribution pasture predicted to occur at 30 arc sec resolution produced by downscaling the coarse grained (0.5°) Land‐use Harmonisation dataset. Colours are ramped light (low) to dark (high).
**Figure S5.** Global distribution of urban land‐use predicted to occur at 30 arc sec resolution produced by downscaling the coarse grained (0.5°) Land‐use Harmonisation dataset. Colours are ramped light (low) to dark (high).
**Figure S6.** Visual comparison of New York region of the USA with true colour landsat imagery (a) and the proportions of each of five land‐uses predicted to occur by the original coarse grained (0.5°) Land‐use Harmonisation dataset (b–f) and the fine grained (30 arc sec) downscaled land‐use datasets (g–k).
**Figure S7.** Visual comparison of part of the Mediterranean including parts of north Africa and Spain with true colour landsat imagery (a) and the proportions of each of five land‐uses predicted to occur by the original coarse grained (0.5°) Land‐use Harmonisation dataset (b–f) and the fine grained (30 arc sec) downscaled land‐use datasets (g–k).
**Figure S8.** Visual comparison of part of south‐east Asia including parts of north Vietnam, Laos and China with true colour landsat imagery (a) and the proportions of each of five land‐uses predicted to occur by the original coarse grained (0.5°) Land‐use Harmonisation dataset (b–f) and the fine grained (30 arc sec) downscaled land‐use datasets (g–k).
**Table S1.** *R* ^2^ values from comparison of initial LUH coarse‐scale (0.5°) data values and the aggregated means of the fine‐grained (30″) downscaled land use data.
**Table S2.** Absolute differences in aggregated proportions of each of the five different land‐uses from the original Land‐use Harmonisation datasets and the new downscaled dataset.
**Figure S9.** Distribution of absolute difference in land‐use predictions calculated in the areas where two neighbouring models provided overlapping predictions.
**Table S3.** Results from Relative Operating Characteristic curve analysis and bootstrapped comparisons of the Area Under the Relative Operating Characteristic curve (AUC) for land‐use predicted from the coarse grained Land‐use Harmonisation (LUH) datasets and the fine grained downscaled land‐uses from the current study.
