# Supplementary material for: Downscaling land‐use data to provide global 30″ estimates of five land‐use classes
Source: Ecol Evol. 2016 Mar 30;6(9):3040–55. doi: 10.1002/ece3.2104 (PMC4814442; doi:10.1002/ece3.2104)
Supplement: Supplementary file 1 — Figure S1. Global distribution of primary habitat predicted to occur at 30 arc sec resolution produced by downscaling the coarse grained (0.5°) Land‐use Harmonisation dataset. Colours are ramped light (low) to dark (high). Figure S2. Global distribution of secondary habitat predicted to occur at 30 arc sec resolution produced by downscaling the coarse grained (0.5°) Land‐use Harmonisation dataset. Colours are ramped light (low) to dark (high). Figure S3. Global distribution of cropland predicted to occur at 30 arc sec resolution produced by downscaling the coarse grained (0.5°) Land‐use Harmonisation dataset. Colours are ramped light (low) to dark (high). Figure S4. Global distribution pasture predicted to occur at 30 arc sec resolution produced by downscaling the coarse grained (0.5°) Land‐use Harmonisation dataset. Colours are ramped light (low) to dark (high). Figure S5. Global distribution of urban land‐use predicted to occur at 30 arc sec resolution produced by downscaling the coarse grained (0.5°) Land‐use Harmonisation dataset. Colours are ramped light (low) to dark (high). Figure S6. Visual comparison of New York region of the USA with true colour landsat imagery (a) and the proportions of each of five land‐uses predicted to occur by the original coarse grained (0.5°) Land‐use Harmonisation dataset (b–f) and the fine grained (30 arc sec) downscaled land‐use datasets (g–k). Figure S7. Visual comparison of part of the Mediterranean including parts of north Africa and Spain with true colour landsat imagery (a) and the proportions of each of five land‐uses predicted to occur by the original coarse grained (0.5°) Land‐use Harmonisation dataset (b–f) and the fine grained (30 arc sec) downscaled land‐use datasets (g–k). Figure S8. Visual comparison of part of south‐east Asia including parts of north Vietnam, Laos and China with true colour landsat imagery (a) and the proportions of each of five land‐uses predicted to occur by the original coarse grained (0.5°) Land [file ECE3-6-3040-s001.docx]

**SUPPLEMENTARY MATERIAL**

Downscaling land-use data to provide global 30” estimates of five land-use classes

Hoskins A J, Bush A, Gilmore J, Harwood T, Hudson L N, Ware C, Williams K J, Ferrier S

**DATA ACCESSIBILITY**

All five globally complete land-use data layers are available via the CSIRO’s Data Access Portal which can be accessed via the link: <http://doi.org/10.4225/08/56DCD9249B224>


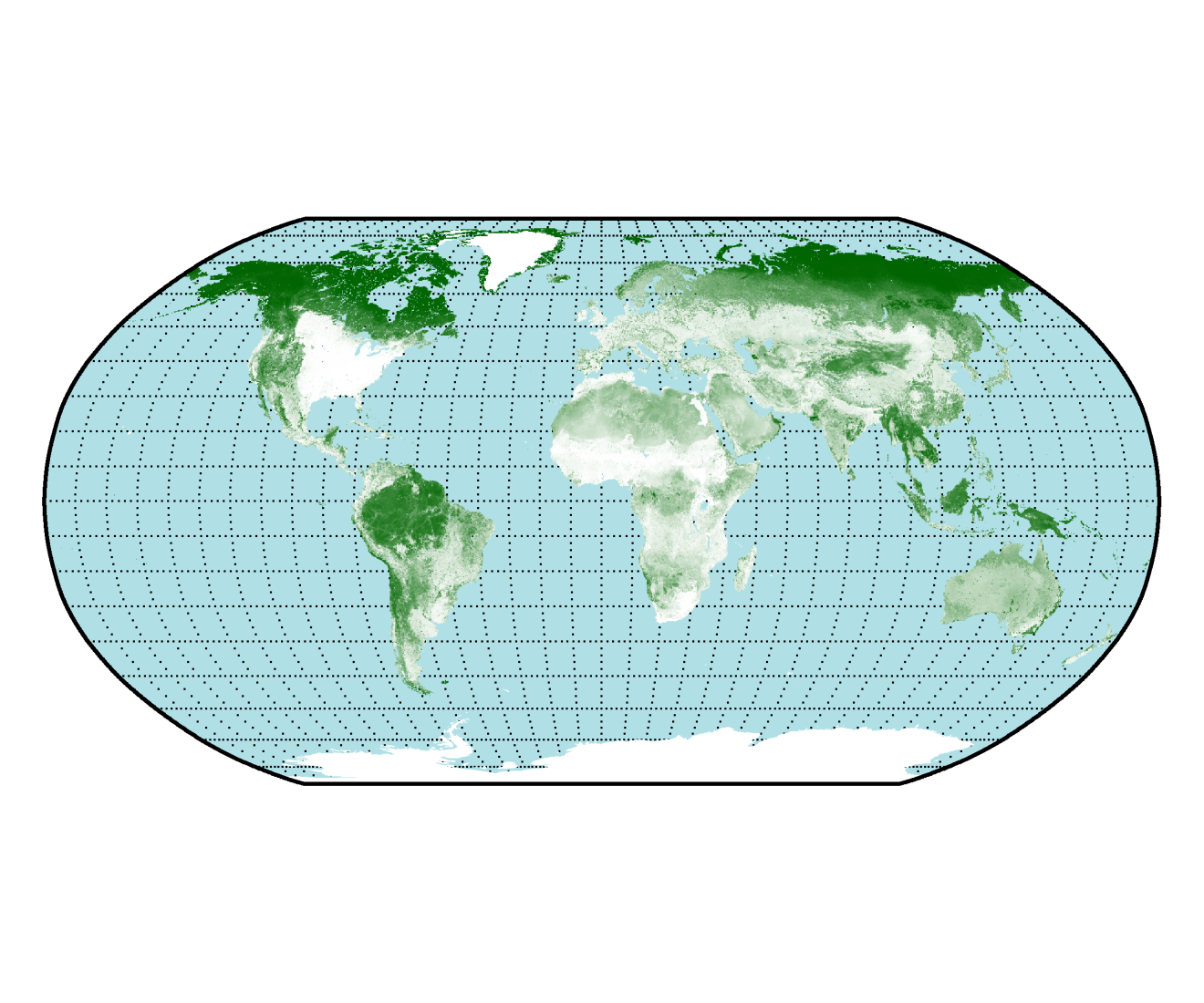


Figure S1: Global distribution of primary habitat predicted to occur at 30 arc sec resolution produced by downscaling the coarse grained (0.5^o^) Land-use Harmonisation dataset. Colours are ramped light (low) to dark (high).


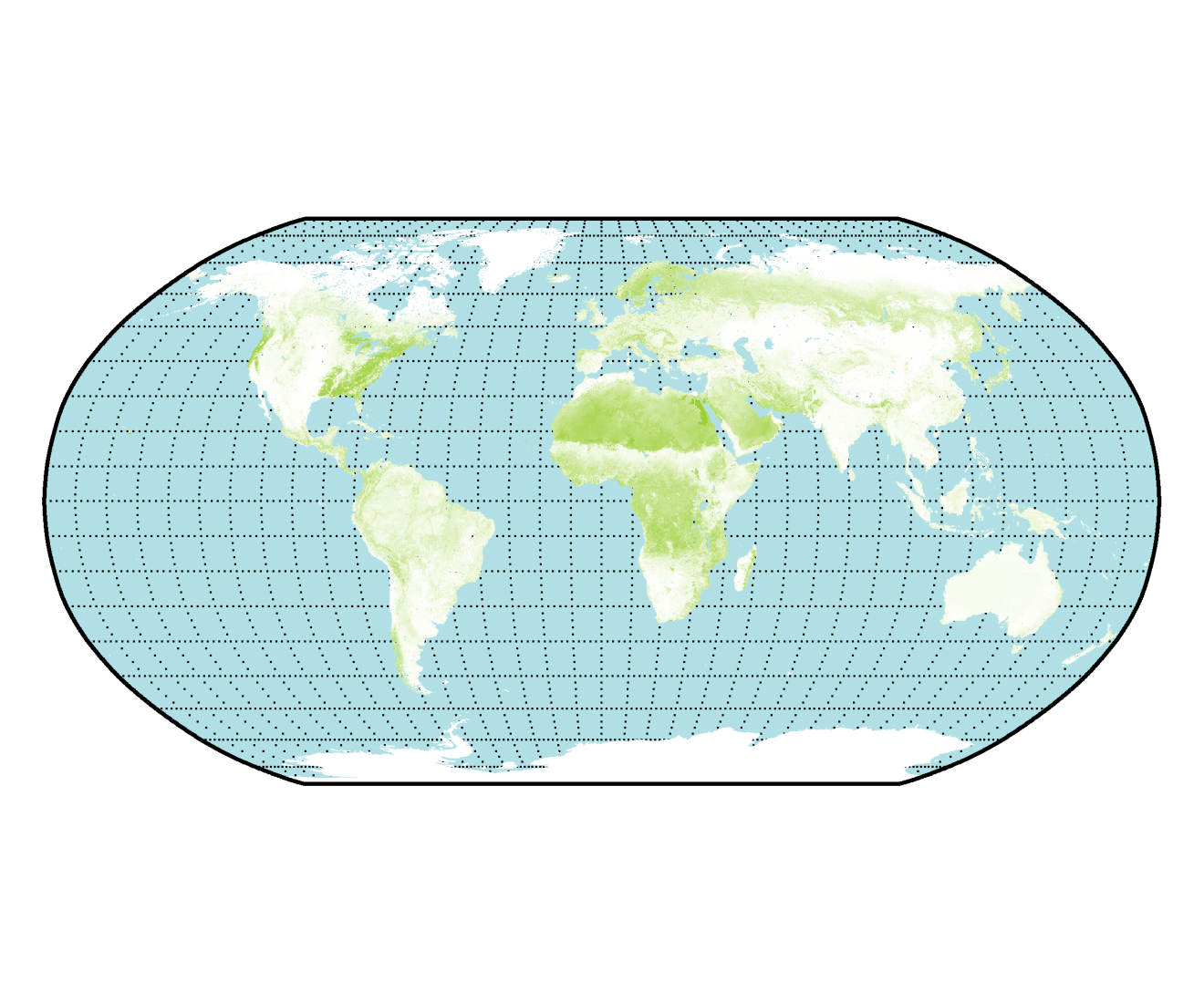


Figure S2: Global distribution of secondary habitat predicted to occur at 30 arc sec resolution produced by downscaling the coarse grained (0.5^o^) Land-use Harmonisation dataset. Colours are ramped light (low) to dark (high).


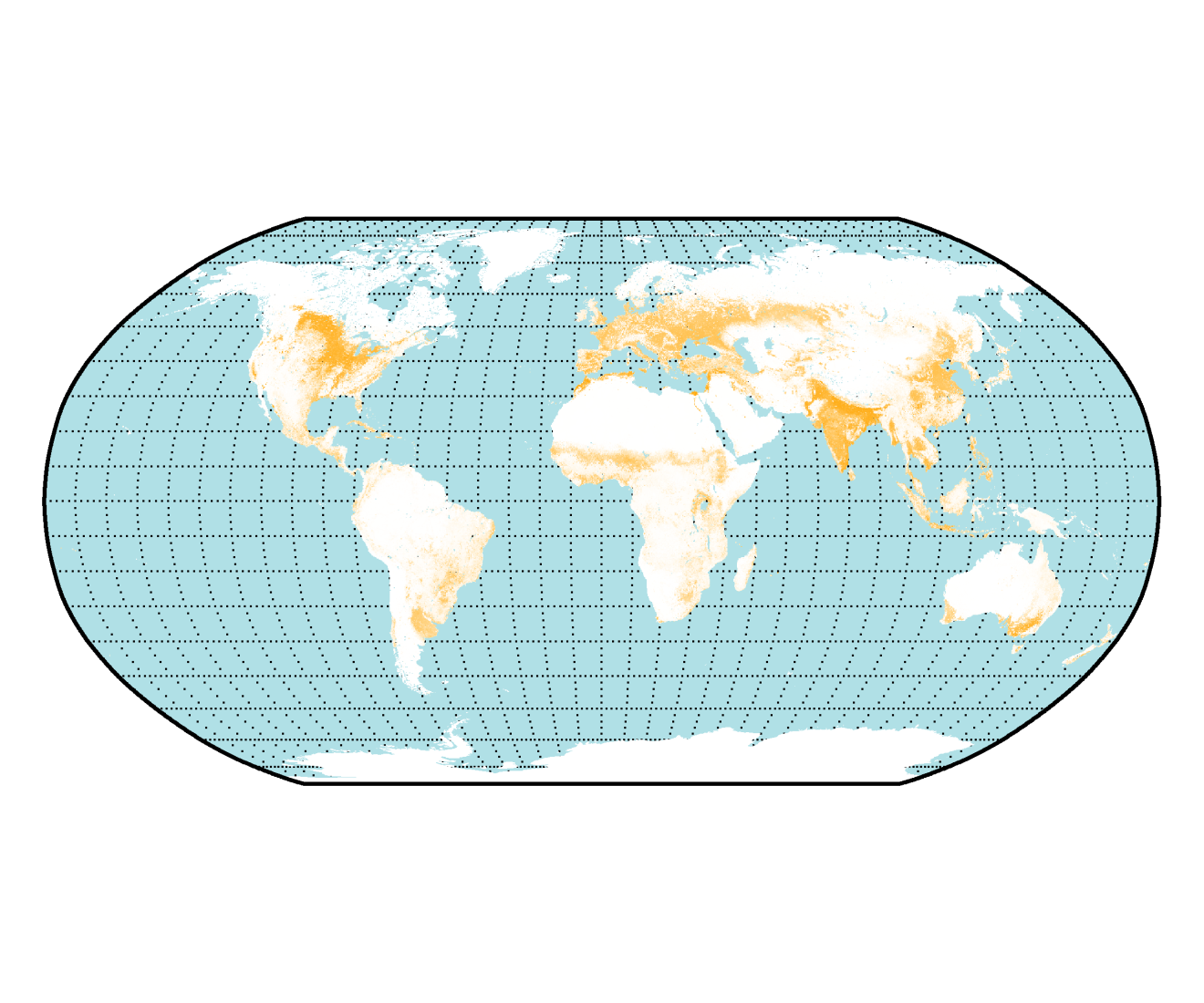


Figure S3: Global distribution of cropland predicted to occur at 30 arc sec resolution produced by downscaling the coarse grained (0.5^o^) Land-use Harmonisation dataset. Colours are ramped light (low) to dark (high).


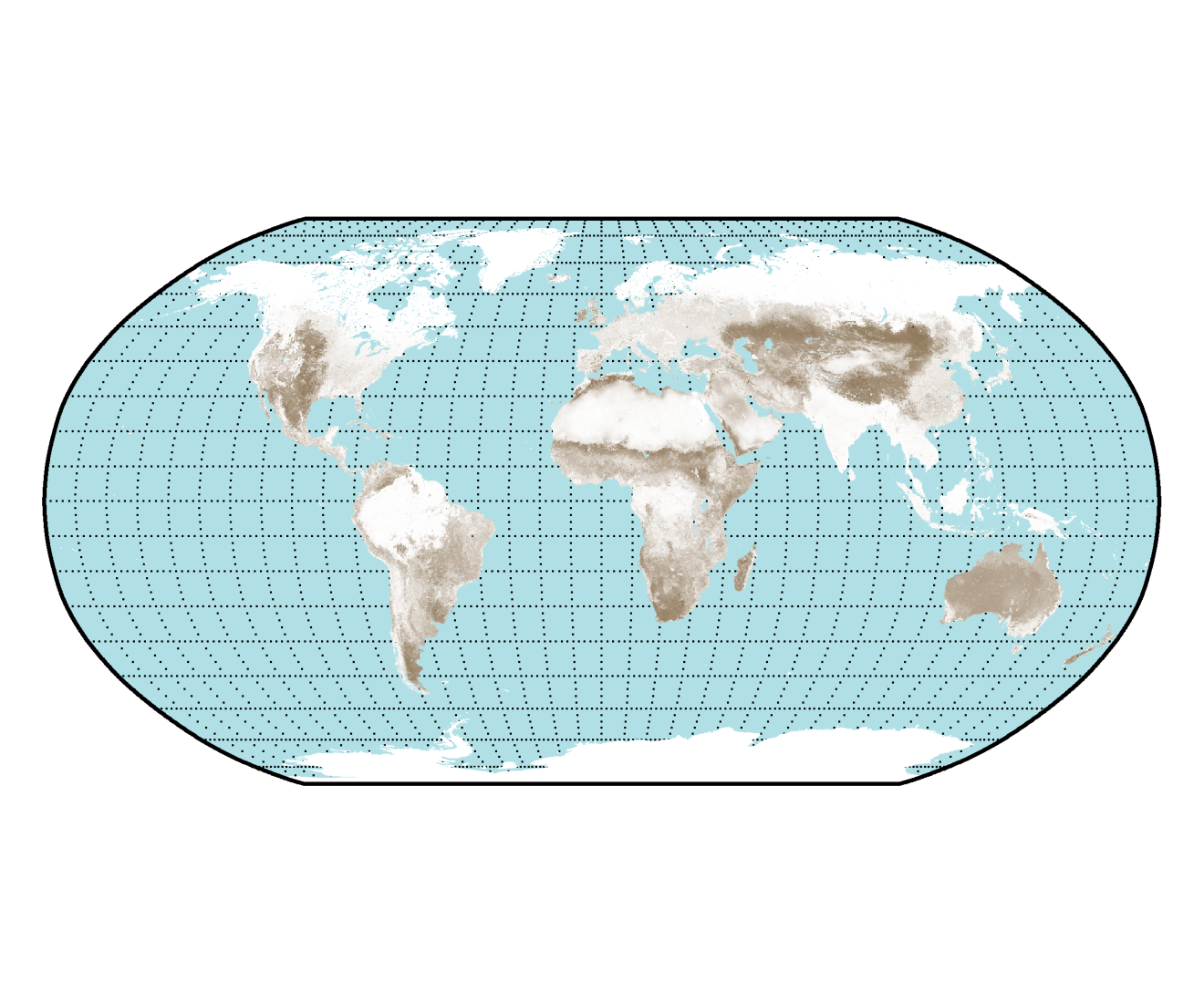


Figure S4: Global distribution pasture predicted to occur at 30 arc sec resolution produced by downscaling the coarse grained (0.5^o^) Land-use Harmonisation dataset. Colours are ramped light (low) to dark (high).


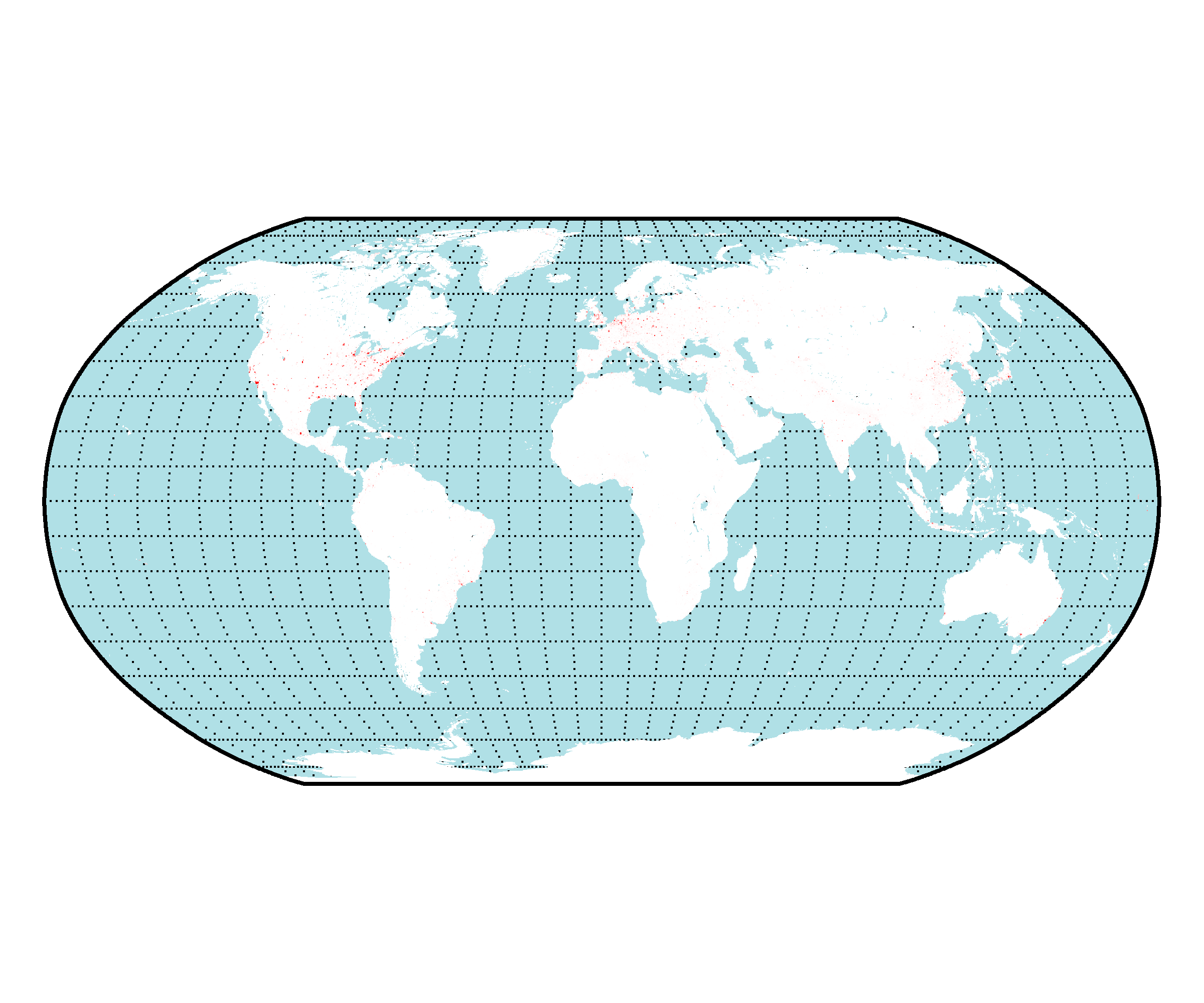


Figure S5: Global distribution of urban land-use predicted to occur at 30 arc sec resolution produced by downscaling the coarse grained (0.5^o^) Land-use Harmonisation dataset. Colours are ramped light (low) to dark (high).


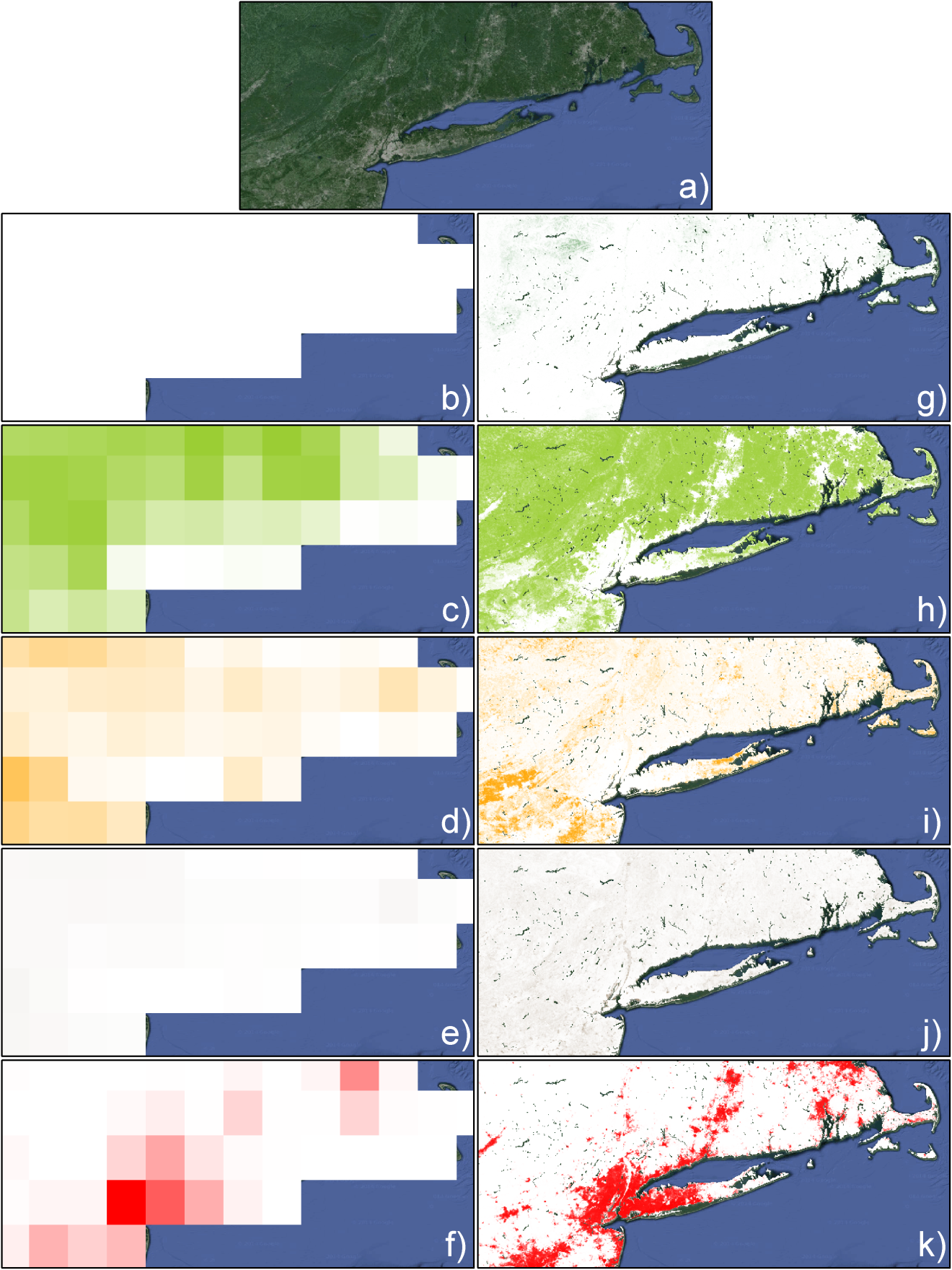


Figure S6: Visual comparison of New York region of the USA with true colour landsat imagery (a) and the proportions of each of five land-uses predicted to occur by the original coarse grained (0.5^o^) Land-use Harmonisation dataset (b - f) and the fine grained (30 arc sec) downscaled land-use datasets (g - k). Colour intensity in panels b - k represent low to high proportions of land-use within a pixel. Each pair of panels in rows 2 - 5 show an individual land use; primary vegetation (b and g), secondary vegetation (c and h), cropping (d and i), pasture (e and j) and urban (f and k).


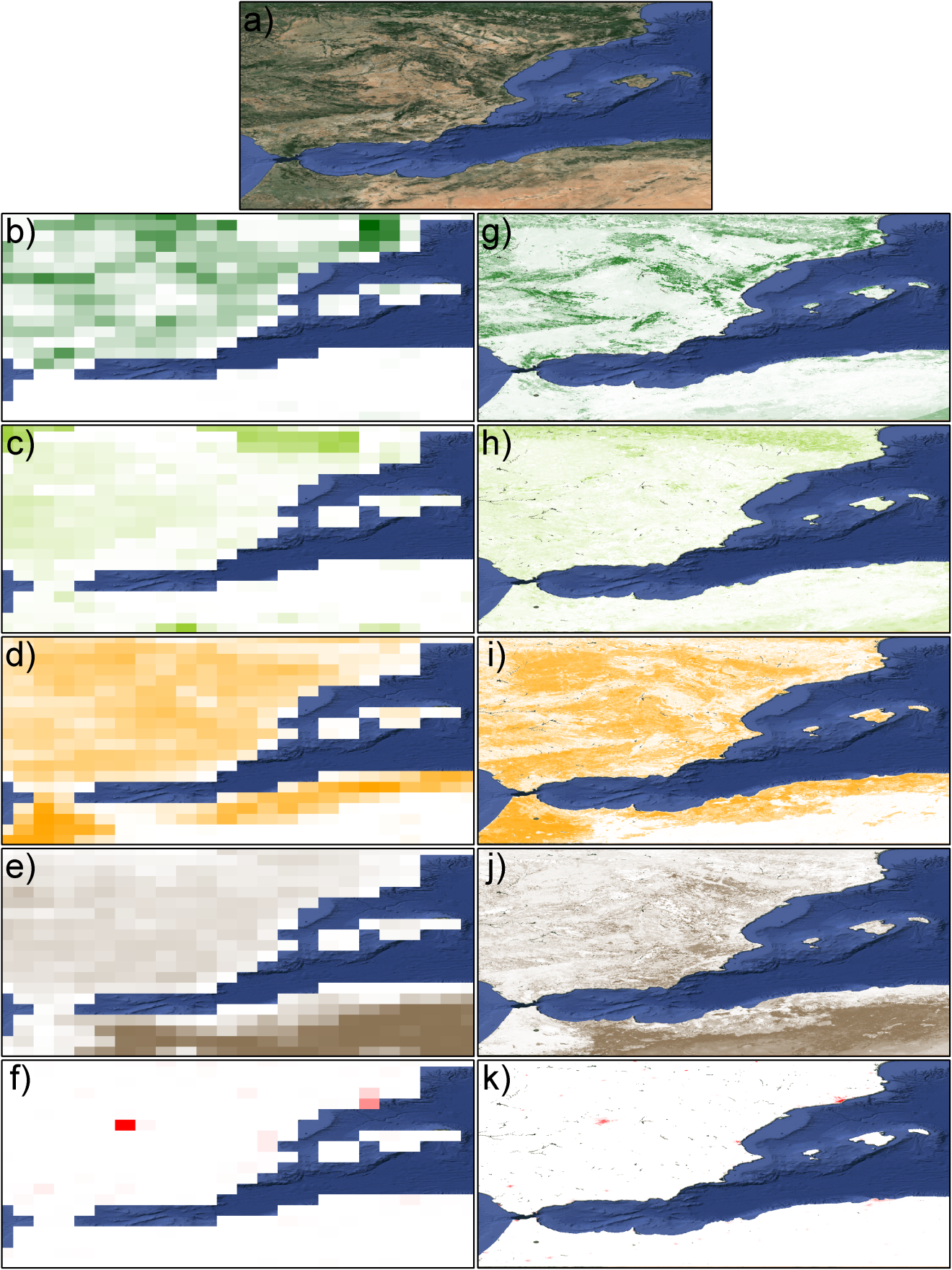


Figure S7: Visual comparison of part of the Mediterranean including parts of north Africa and Spain with true colour landsat imagery (a) and the proportions of each of five land-uses predicted to occur by the original coarse grained (0.5^o^) Land-use Harmonisation dataset (b - f) and the fine grained (30 arc sec) downscaled land-use datasets (g - k). Colour intensity in panels b - k represent low to high proportions of land-use within a pixel. Each pair of panels in rows 2 - 5 show an individual land use; primary vegetation (b and g), secondary vegetation (c and h), cropping (d and i), pasture (e and j) and urban (f and k).


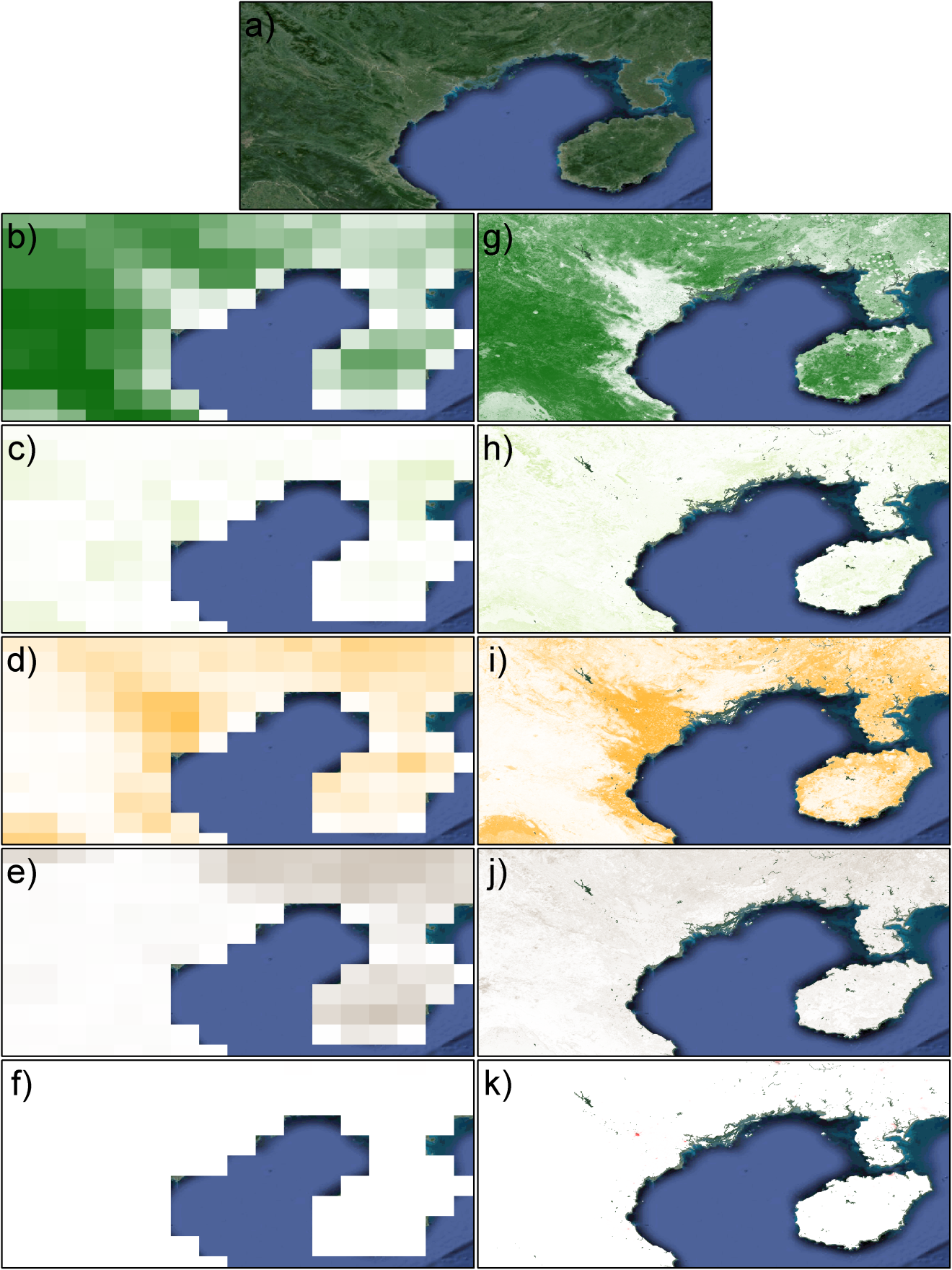


Figure S8: Visual comparison of part of south-east Asia including parts of north Vietnam, Laos and China with true colour landsat imagery (a) and the proportions of each of five land-uses predicted to occur by the original coarse grained (0.5^o^) Land-use Harmonisation dataset (b - f) and the fine grained (30 arc sec) downscaled land-use datasets (g - k). Colour intensity in panels b - k represent low to high proportions of land-use within a pixel. Each pair of panels in rows 2 - 5 show an individual land use; primary vegetation (b and g), secondary vegetation (c and h), cropping (d and i), pasture (e and j) and urban (f and k).

Table S1: *R^2^* values from comparison of initial LUH coarse-scale (0.5^o^) data values and the aggregated means of the fine-grained (30”) downscaled land use data

|  | **Globe** | **AA** | **AT** | **IM** | **NA** | **NT** | **OC** | **PA** | **Mean** |
| --- | --- | --- | --- | --- | --- | --- | --- | --- | --- |
| **Primary** | 0.59 ± 0.25 | 0.69 ± 0.13 | 0.42 ± 0.35 | 0.66 ± 0.26 | 0.66 ± 0.26 | 0.59 ± 0.17 | 0.47 ± 0.07 | 0.48 ± 0.17 | **0.57 ± 0.21** |
| **Secondary** | 0.52 ± 0.23 | 0.41 ± 0.11 | 0.47 ± 0.21 | 0.58 ± 0.18 | 0.65 ± 0.28 | 0.45 ± 0.22 | 0.33 ± 0.19 | 0.64 ± 0.2 | **0.51 ± 0.2** |
| **Pasture** | 0.74 ± 0.16 | 0.8 ± 0.17 | 0.76 ± 0.19 | 0.68 ± 0.18 | 0.74 ± 0.23 | 0.65 ± 0.16 | 0.43 ± 0.02 | 0.7 ± 0.15 | **0.69 ± 0.16** |
| **Cropping** | 0.78 ± 0.14 | 0.79 ± 0.18 | 0.73 ± 0.12 | 0.86 ± 0.08 | 0.86 ± 0.1 | 0.77 ± 0.11 | 0.77 ± 0.2 | 0.76 ± 0.2 | **0.79 ± 0.14** |
| **Urban** | 0.76 ± 0.18 | 0.77 ± 0.2 | 0.67 ± 0.18 | 0.72 ± 0.19 | 0.77 ± 0.22 | 0.79 ± 0.16 | 0.63 ± 0.01 | 0.76 ± 0.13 | **0.73 ± 0.16** |
| **Mean** | **0.68 ± 0.19** | **0.69 ± 0.16** | **0.61 ± 0.21** | **0.7 ± 0.18** | **0.74 ± 0.22** | **0.65 ± 0.16** | **0.52 ± 0.1** | **0.67 ± 0.17** |  |

Table S2: Absolute differences in aggregated proportions of each of the five different land-uses from the original Land-use Harmonisation datasets and the new downscaled dataset

|  | **Globe** | **Australasia** | **Afrotropics** | **Indo-Malaysia** | **Nearctic** | **Neotropics** | **Oceania** | **Palearctic** |
| --- | --- | --- | --- | --- | --- | --- | --- | --- |
| **Primary** | 0.0086 | 0.0087 | 0.0013 | 0.0011 | 0.0149 | 0.0097 | 0.0969 | 0.0093 |
| **Secondary** | 0.0029 | 0.0011 | 0.0045 | 0.0036 | 0.0078 | 0.0059 | 0.1158 | 0.003 |
| **Cropping** | 0.0021 | 0.0004 | 0.0029 | 0.0009 | 0.0052 | 0.0015 | 0.0035 | 0.0011 |
| **Pasture** | 0.0041 | 0.0101 | 0.0024 | 0.0036 | 0.0068 | 0.002 | 0.0418 | 0.005 |
| **Urban** | 0.0006 | 0.0000 | 0.0004 | 0.0003 | 0.0049 | 0.0003 | 0.0642 | 0.0002 |


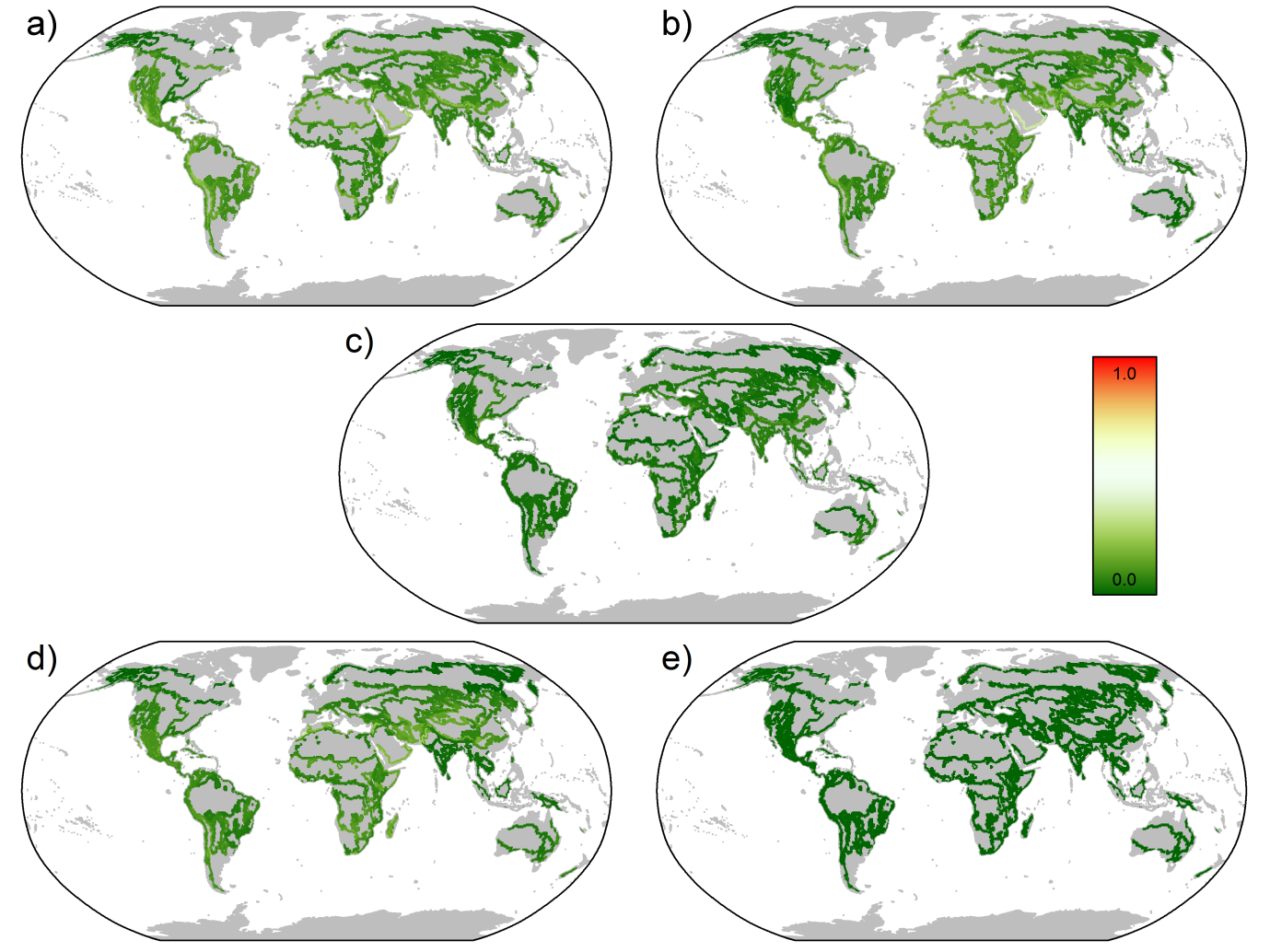


Figure S9: Distribution of absolute difference in land-use predictions calculated in the areas where two neighbouring models provided overlapping predictions. a) Primary habitat, b) Secondary habitat, c) Cropping, d) Pasture, and e) Urban.

Table S3: Results from Relative Operating Characteristic curve analysis and bootstrapped comparisons of the Area Under the Relative Operating Characteristic curve (AUC) for land-use predicted from the coarse grained Land-use Harmonisation (LUH) datasets and the fine grained downscaled land-uses from the current study.

|  | **Downscaled AUC** | **LUH AUC** | **Δ AUC** | **D** | ***p*** |
| --- | --- | --- | --- | --- | --- |
| **Cropping** | 0.79 | 0.71 | 0.08 | 9.3143 | < 0.000001 |
| **Pasture** | 0.76 | 0.73 | 0.03 | 5.7825 | < 0.000001 |
| **Primary** | 0.73 | 0.64 | 0.09 | 10.1773 | < 0.000001 |
| **Secondary** | 0.59 | 0.47 | 0.12 | 3.7728 | 0.000161 |
| **Urban** | 0.98 | 0.95 | 0.03 | 6.0309 | < 0.000001 |
